# Assessment of Anti-SARS-CoV-2 Antibodies Post-Coronavac Vaccination in the Amazon Region of Brazil

**DOI:** 10.3390/vaccines9101169

**Published:** 2021-10-12

**Authors:** Carlos David Araújo Bichara, Maria Alice Freitas Queiroz, Ednelza da Silva Graça Amoras, Gergiane Lopes Vaz, Izaura Maria Vieira Cayres Vallinoto, Cléa Nazaré Carneiro Bichara, Isabella Pinheiro Costa do Amaral, Ricardo Ishak, Antonio Carlos Rosário Vallinoto

**Affiliations:** 1 Laboratory of Virology, Institute of Biological Sciences, Federal University of Pará (UFPA), Belém 66075-110, PA, Brazil; bichara@amaralcosta.com.br (C.D.A.B.); alicefarma@hotmail.com (M.A.F.Q.); ednelza@hotmail.com (E.d.S.G.A.); ivallinoto@ufpa.br (I.M.V.C.V.); rishak@ufpa.br (R.I.); 2 Amaral Costa Diagnostic Medicine, Belém 66055-050, PA, Brazil; gergiane@amaralcosta.com.br (G.L.V.); isabella@amaralcosta.com.br (I.P.C.d.A.); 3 Graduate Program in Biology of Infectious and Parasitic Agents, Institute of Biological Sciences, Federal University of Pará (UFPA), Belém 66075-110, PA, Brazil; 4 Center for Biological and Health Sciences, School of Medicine, Pará State University (UEPA), Belém 66087-870, PA, Brazil; cleacarneirobichara@gmail.com

**Keywords:** COVID-19, SARS-CoV-2, vaccine, Coronavac, antibody

## Abstract

The present study evaluated the frequency of seropositivity for anti-SARS-CoV-2 (S1 and S2) total antibodies and anti-SARS-CoV-2 (receptor binding domain-RBD-S1) neutralizing antibodies in individuals vaccinated with the immunizing agent Coronavac. This was a cross-sectional study involving 358 individuals divided into two groups. Group 1 consisted of 205 volunteers who were tested for anti-SARS-CoV-2 total antibodies; group 2 consisted of 153 individuals tested for the presence of anti-SARS-CoV-2 neutralizing antibodies. Seropositivity was greater than 70% in both groups, although 17.6% and 20.9% of individuals showed no neutralizing or total antibody reactivity, respectively. The frequency of anti-SARS-CoV-2 total antibodies displayed a significantly different distribution between the sexes but not according to age. The frequency of anti-SARS-CoV-2 neutralizing antibodies was 93.3% (95% CI 68.1–99.8) in the age group from 21 to 40 years but significantly decreased with advancing age, and was 76.2% (95% CI 52.8–91.8) for 41 to 60 years, 72.5% (95% CI 62.8–80.9) for 61 to 80 years, and 46.7% (95% CI 21.3–73.4) for >80 years. Our results reveal a high prevalence of anti-SARS-CoV-2 total antibodies and anti-SARS-CoV-2 neutralizing antibodies in individuals who received both doses of the Coronavac vaccine, suggesting a lower effectiveness of the humoral immune response among those older than 60 years of age, which might be associated with senescence of the immune system.

## 1. Introduction

The severe acute respiratory syndrome coronavirus 2 (SARS-CoV-2) pandemic that emerged in Wuhan, China in November 2019 [1] was accompanied by a series of concerns, questions, and lessons [2]. The conditions brought about by this virus triggered the most intensive race in the history of science worldwide to develop a vaccine capable of eliciting neutralizing antibodies against SARS-CoV-2 and reinfections by different variants of the virus [3], conferring protection against severe cases of coronavirus disease 2019 (COVID-19) [4]. New anti-SARS-CoV-2 vaccine development platforms using the following—inactivated virus, nonreplicating viral vector, subunit, viral-like particle, DNA, and mRNA—were implemented [5,6], generating hundreds of records of preclinical and phase II, III, and IV clinical studies [7]. Along with proposals for new technologies for the creation of anti-SARS-CoV-2 vaccines, there were also doubts and concerns [8], especially regarding the safety and efficacy of the new platforms, which, until then, had not been applied to humans [9].

Parallel to advances in our understanding of the immunological mechanisms present in SARS-CoV-2 infection and in the modulation of COVID-19, phase II and III studies showed satisfactory and promising efficacy and safety results for different anti-SARS-CoV-2 vaccine platforms [5]. This has resulted in 14 vaccines that are in use in different countries thus far.

Zhang et al. [10] investigated the immunogenic characteristics of different vaccine platforms and reported that humoral and cellular immune responses differed when administered individually, with inactivated vaccines showing lower levels of neutralizing antibody and T cell responses. A preprint of a retrospective cohort study assessed the effectiveness of Vaxzevria and Coronavac vaccines for COVID-19 in Brazil and reported overall effectiveness against severe COVID-19 for Vaxzevria up to 89 years of age and for Coronavac up to 79 years of age [11].

In Brazil, the National Health Surveillance Agency (ANVISA), a government agency responsible for regulating pharmacological and immunobiological inputs, approved the definitive registration of the Pfizer and AstraZeneca vaccines in January 2021. Coronavac (Sinovac) and Janssen-CILAG vaccines have been approved only for emergency use [12].

The main question and reason for the different opinions and discussions is associated with the effectiveness of mass vaccination campaigns, especially with regard to the effectiveness of immunization in generating protective immunity and immunological memory. This dilemma highlights the importance of evaluating variables such as sex and age, as the immune response can exhibit different dynamics based on sex [13] and physiological senescence of the immune system with age [14]. In this context, serological studies have been carried out to assess the effectiveness of generating post-vaccination neutralizing antibodies at the population level [15].

The present study examined the frequency of anti-SARS-CoV-2 total antibodies specific to the S1 and S2 portions of the viral spike protein, as well as the presence of anti-SARS-CoV-2 (receptor binding domain (RBD-S1)) neutralizing antibodies, in two independent groups of individuals who sought care at the Amaral Costa Medicina Diagnóstica laboratory after receiving the second dose of the Coronavac vaccine.

## 2. Materials and Methods

### 2.1. Studied Samples

This was a cross-sectional study in which blood samples were collected from March to April 2021 and included 358 individuals (Table 1) of both sexes (138 males and 220 females) aged between 21 and 96 years (average 66.6 years). The persons involved in the study voluntarily sought care at the Amaral Costa Medicina Diagnóstica in the city of Belem, the capital of the State of Para (Northern Brazil), after their second dose of Coronavac (Sinovac Research and Development Co. Ltd., Haidian District, Beijing, China/Butantan, São Paulo, Brazil) for the purposes of confirming serological conversion. Those persons who presented evidence of previous vaccination (second dose) within 30 days were invited to participate in the study. The vaccination regimen adopted was two doses with a time interval of 20 days. Of the total number of individuals analyzed, we performed an anti-SARS-CoV-2 total antibody test (S1 and S2) for 205; 153 individuals were tested for the presence of anti-SARS-CoV-2 neutralizing antibodies (RBD-S1).

This project was submitted to and approved by the Human Research Ethics Committee of the Institute of Health Sciences of the Federal University of Pará (CAAE: 31800720.1.0000.0018) in compliance with the guidelines and regulatory standards for research involving human beings. Individuals who agreed to participate in the study signed an informed consent form.

### 2.2. Ethical Aspects

The study was approved by the Ethics and Research in Human Beings Committee of the Health Sciences Institute of the Federal University of Pará (CAAE: 31800720.1.0000.0018) in compliance with the guidelines and regulatory standards for research involving human beings, in accordance with the Declaration of Helsinki.

### 2.3. Antibody Analysis

Whole blood samples (5 mL) were collected in vacuum tubes without anticoagulant. Serum was separated by centrifugation. Investigation of anti-SARS-CoV-2 total antibodies (S1 and S2) was performed using a qualitative microparticle chemiluminescent immunoassay (CMIA) with the LIAISON**^®^** XL Analyzer automated platform (DiaSorin, Saluggia, Italy) following the manufacturer’s recommendations. The reference ranges were non-reagent (<12 AU/mL), indeterminate (12.0 ≥ x < 15.0 UA/mL), and reagent (>15.0 AU/mL).

Anti-SARS-CoV-2 (RBD-S1) neutralizing antibodies were detected using the competitive enzyme immunoassay GenScript cPass™ SARS-CoV-2 Neutralization Antibody Detection kit (GenScript, Piscataway, New Jersey, USA) following the manufacturer’s protocol. The approach, also known as the SARS-CoV-2 Surrogate Virus Neutralization Test (sVNT) kit, is a faster, easier, more scalable, and automatable alternative to traditional neutralizing antibody tests, such as the virus neutralization test (VNT), pseudovirus neutralization test (pVNT), and plaque reduction neutralization test (PRNT). The reference ranges used were non-reagent (<20%), indeterminate (20 ≥ x ≤ 29%), and reagent (>30%).

### 2.4. Statistical Analysis

Information on sex, age, and antibody status was tabulated in Excel software. The calculation of antibody frequencies was performed by direct counting, and the significance of comparisons between groups was assessed by chi-square and G tests [16] using the Bioestat version 5.3 program. Differences were considered statistically significant when the *p*-value was <0.05.

## 3. Results

When evaluating anti-SARS-CoV-2 total and neutralizing antibodies, 270 samples (75.4%) presented positive results, 70 (19.6%) were non-reagent, and 18 (5.0%) were indeterminate (Table 1).

Testing for anti-SARS-CoV-2 total antibodies (205 individuals) showed a positive result for 159 samples (77.6%), while 43 (20.9%) were non-reagent and 3 (1.5%) were indeterminate (Table 1). Regarding anti-SARS-CoV-2 neutralizing antibodies (153 individuals tested), 111 samples (72.6%) presented a positive result, 27 (17.6%) were non-reagent, and 15 (9.8%) were indeterminate (Table 1).

The numbers of individuals according to sex in each age group were as follows: 21–40 years (F = 26 (74.3%) and M = 9 (25.7%)), 41–60 years (F = 36 (60%) and M = 24 (40%)), 61–80 years (F = 129 (58.3%) and M = 92 (41.7%)), and > 80 years (F = 29 (69%) and M = 13 (31%)).

**Figure 1:**
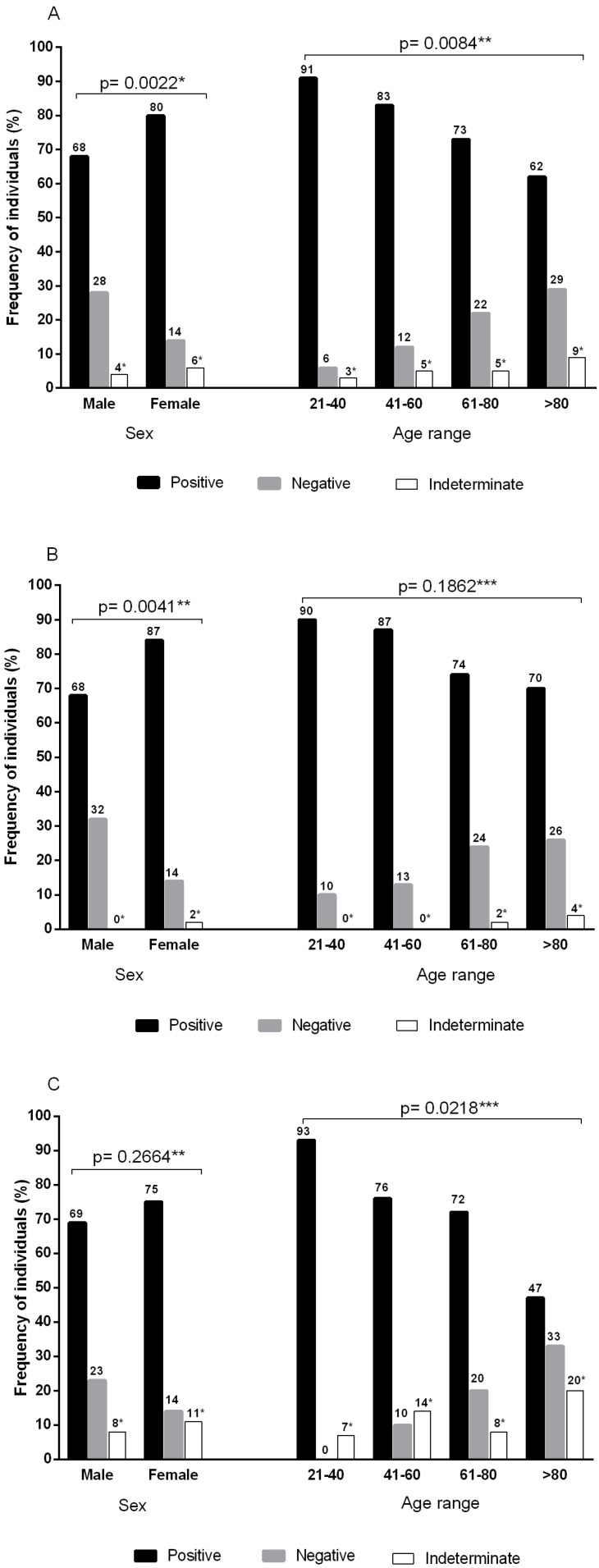
Frequencies of anti-SARS-CoV-2 antibodies according to sex and age group. (**A**) Pooled frequencies of anti-SARS-CoV-2 total antibodies (S1/S2) plus anti-SARS-CoV-2 IgG neutralizing antibodies (RBD-S1). Sample size by sex: male (*n* = 138) and female (*n* = 220). Sample size by age group: 21–40 (*n* = 35), 41–60 (*n* = 60), 61–80 (*n* = 221), and >80 (*n* = 42). (**B**) Frequencies of total anti-SARS-CoV-2 antibodies (S1/S2). Sample size by sex: male (*n* = 77) and female (*n* = 128). Sample size by age group: 21–40 (*n* = 20), 41–60 (*n* = 39), 61–80 (*n* = 119), and >80 (*n* = 27). (**C**) Frequencies of neutralizing IgG anti-SARS-CoV-2 (RBD-S1) antibodies. Sample size by sex: male (*n* = 61) and female (*n* = 92). Sample size by age group: 21–40 (*n* = 15), 41–60 (*n* = 21), 61–80 (*n* = 102), and >80 (*n* = 15). * Indeterminate results were not included in the statistical analysis; ** chi-square test; *** G test.

The seropositivity profiles (reagent vs. non-reagent) according to the results of both tests revealed a significantly higher value (*p* = 0.0022) in females (80%) than in males (68%; Figure 1A). A similar result was observed (87% vs. 68%; *p* = 0.0041) for the 205 individuals who underwent testing for anti-SARS-CoV-2 total antibodies (Figure 1B). However, no significant differences between sexes were found for the frequency of anti-SARS-CoV-2 neutralizing antibodies (75% vs. 69%; Figure 1C).

The seropositivity profile according to the results of anti-SARS-CoV-2 total antibodies plus anti-SARS-CoV-2 neutralizing antibodies (Figure 1A) indicated significant differences from the pooled analysis of age groups (*p* = 0.0084). The highest frequency occurred in the age group of 21–40 years (91.4%; 95% CI 76.9–98.2), gradually decreasing as age increased to 83.3% (95% CI 71.5–91.7) for 41–60 years, 73.9% (95% CI 65.1–81.6) for 61–80 years, and 61.9% (95% CI 45.6–76.4) for >80 years. These significant differences were not observed when measuring anti-SARS-CoV-2 total antibodies (Figure 1B) but followed the same pattern when measuring only anti-SARS-CoV-2 neutralizing antibodies (*p* = 0.0218; Figure 1C); individuals aged 21 to 40 years showed 93.3% (95%CI 68.1–76.2) seropositivity, which decreased gradually with age to 76.2% (95%CI 52.8–91.8) for 41 to 60 years, 72.5% (95%CI 62.8–80.9) for 61 to 80 years, and 46.6% (95%CI 21.3–73.4) for >80 years.

## 4. Discussion

The prevalence of seropositivity for anti-SARS-CoV-2 total antibodies and anti-SARS-CoV-2 neutralizing antibodies was evaluated in the present study in individuals vaccinated with two doses of Coronavac. The results were similar, regardless of the method used to assess humoral immunological response, including the frequency of those who did not produce antibodies. However, seropositivity values were lower than those reported by the manufacturer of the immunizing agent during phase I and II randomized clinical trials in adults, young people, and elderly people over 60 years, and were higher than the value of vaccine efficacy reported by health care professionals in direct contact with COVID-19 patients [17]. A limitation of the present study is the lack of information on the occurrence of previous infection in vaccinated individuals, a variable that might interfere with the assessment of post-vaccination seroconversion.

Recent studies have shown that the Coronavac vaccine is efficient in eliciting neutralizing antibodies [18,19,20,21], which together with the present results, particularly those obtained for anti-SARS-CoV-2 neutralizing antibodies, are encouraging. Indeed, considering the percentage of positivity observed in the present study, the findings suggest that mass vaccination of the population with Coronavac can generate collective protection [22].

Overall, there are different opinions and discussions about the efficacy and efficiency of immunizations with anti-SARS-CoV-2 vaccines in relation to the potential for generating protective immunity and the persistence of immunological memory [9,23], especially with regard to variables such as sex and age. In general, the immune response exhibits distinct dynamics based on factors such as sex [13,24] and immune system senescence [14]. In the present study, the frequency of anti-SARS-CoV-2 total antibodies was significantly higher in females, which corroborates the literature describing females as presenting increased inflammatory and humoral responses to COVID-19 [14], but we do not rule out the possibility that this result is due to a sampling bias.

Furthermore, with regard to seropositivity for anti-SARS-CoV-2 neutralizing antibodies, a high prevalence was observed among young adults; the lowest frequency was detected among elderly individuals, which suggests a lower effectiveness of the vaccine to stimulate the humoral immune response in the elderly. Despite the small sample size investigated herein, which can be a limitation of our study, our results seem to support evidence for a functional and progressive decline in the immune system in elderly patients [14]. Nevertheless, it is noteworthy that a recent phase I/II clinical trial study demonstrated immunogenicity after Coronavac vaccination in adults aged 60 years and older as well as its safety and tolerability [21]. It is important to emphasize that a lack of post-vaccination humoral immune response detection does not indicate the absence of immunity to SARS-CoV-2, as the serological methods used do not assess the presence of cellular immunity (CD4^+^ and CD8^+^ T lymphocytes), which may occur even in the absence of antibodies [25,26].

## 5. Conclusions

The results presented herein demonstrate a similar pattern in the frequency of seropositivity for anti-SARS-CoV-2 total antibodies and anti-SARS-CoV-2 neutralizing antibodies in individuals who received two doses of the Coronavac vaccine. However, a lower effectiveness of the humoral immune response among the elderly was found, which may be associated with senescence of the immune system. It is important that this result is confirmed in follow-up studies because a third-dose booster might be necessary for this group, especially due to their greater vulnerability to the most severe clinical outcome of COVID-19.

Finally, considering the emergence of virus variants, the neutralizing antibody response after vaccination should be monitored. Our results support the execution of population-based serological studies aimed at better understanding the efficacy of vaccines approved for use in Brazil in terms of their ability to generate herd immunity against SARS-CoV-2.

## Figures and Tables

**Table 1:** Frequency of anti-SARS-CoV-2 total antibodies (S1 and S2) and anti-SARS-CoV-2 neutralizing antibodies (RBD-S1) after two doses of Coronavac in Belem, Para.

Sample	Vaccine	Test	N	Male	Female	Age (mean/SD)	Reagent (%)	Indeterminate (%)	Negative (%)
Group 1	Coronavac	Total antibodies	205	77	128	65.5/14.8	159 (77.6%)	03 (1.5%)	43 (20.9%)
Group 2	Coronavac	Neutralizing antibodies	153	61	93	65.4/14.6	111 (72.6%)	15 (9.8%)	27 (17.6%)
Groups 1 and 2	Coronavac	Total and neutralizing antibodies	358	138	220	65.4/14.7	270 (75.4%)	18 (5.0%)	70 (19.6%)

## Data Availability

Data are available upon request from the corresponding author.
